# The severity of portal hypertension by a non-invasive assessment: acoustic structure quantification analysis of liver parenchyma

**DOI:** 10.1186/s12880-022-00817-2

**Published:** 2022-05-12

**Authors:** Wen-Bin Cai, Ji-Kai Yin, Qiao-ying Li, Yi-Lin Yang, Yun-You Duan, Li Zhang

**Affiliations:** 1 Department of Ultrasound Diagnosis, Tangdu Hospital, The Fourth Military Medical University, Xin Si Road, Ba Qiao District, Xi’an, China; 2 Department of General Surgery, Tangdu Hospital, The Fourth Military Medical University, Xi’an, China; 3 Department of Ultrasound Diagnostics, General Hospital of Tibet Military Region, Lhasa, China

**Keywords:** Acoustic, Ultrasound, Portal hypertension, Gastroesophageal varices, Noninvasive

## Abstract

**Background:**

Acoustic structure quantification (ASQ) has been applied to evaluate liver histologic changes by analyzing the speckle pattern seen on B-mode ultrasound. We aimed to assess the severity of portal hypertension (PHT) through hepatic ultrasonography.

**Methods:**

Sixty patients diagnosed with PHT and underwent surgical treatment with portosystemic shunts were enrolled. Portal pressure (PP) was measured intraoperatively. Patients were divided into subgroups according to the severity of gastroesophageal varices and Child–Pugh class. Three difference ratio (C_m_^2^) values on ASQ histogram mode were analyzed for their relationships with PP, degree of gastroesophageal varices and Child–Pugh liver function. Thirty healthy volunteers matched with the patients for gender and age were enrolled as controls. Comparisons among groups and correlation of the parameters with PP were analyzed. Area under the receive operating characteristic curve was used to evaluate the predicting value of ASQ parameters.

**Results:**

In the patients, the ASQ parameters peak *C*_m_^2^ (*C*_m_^2^_max_), mean *C*_m_^2^ (*C*_m_^2^_mean_) and the highest occurred *C*_m_^2^ value of the obtained red curve (*R*_max_*C*_m_^2^) were all greatly increased (*P* < 0.0001, *P* < 0.0001, *P* = 0.027). Multiple comparisons indicated that, regardless of Child–Pugh class and degree of gastroesophageal varices, the patients had significantly increased *C*_m_^2^_max_ and *C*_m_^2^_mean_ compared with the controls (all *P* < 0.0001). No differences among subgroups were observed. *C*_m_^2^_max_ was significantly statistically correlated with PP (*r* = 0.3505, *P* < 0.01), degree of varices (*r* = 0.4998, *P* < 0.0001). Youden’s index for *C*_m_^2^_max_ with a cut-off value of 140.3 for predicting the presence of PHT, gastroesophageal varices and liver function equal to or worse than Child–Pugh class B were 0.8, 0.91 and 0.84, respectively.

**Conclusions:**

ASQ analysis of ultrasonographic images may have a role in the evaluation of the severity of PHT by detecting liver histologic changes in the speckle pattern caused by cirrhosis.

**Supplementary Information:**

The online version contains supplementary material available at 10.1186/s12880-022-00817-2.

## Introduction

Variceal bleeding due to gastroesophageal varices is associated with high mortality in patients with portal hypertension (PHT) [1]. Preventive surgery is recommended and the selection of optimal candidates is based on an assessment of liver function and portal pressure (PP). Thus, measurement of PP is important in patients with suspected PHT. Hepatic venous pressure gradient (HVPG) is considered the gold standard for PHT assessment in patients with cirrhosis [2–5], but the invasiveness and need for expertise in performing the examination limit its use in routine clinical practice. It would be helpful to have noninvasive methods capable of predicting, with acceptable diagnostic accuracy, the severity of clinically significant PHT.

Many methods have been proposed for assessing patients with PHT, especially to detect those with a high risk of variceal bleeding. Based on the dynamic changes of PHT [6], Doppler parameters have been suggested as a substitute for the invasive measurement of HVPG [7–9]. Due to the “static component” of PHT—a mechanical consequence of the associated hepatic architectural disorder—research has focused on analyzing cirrhotic liver parenchyma by ultrasound. Methods include liver stiffness measurement by means of transient elastography (TE) [10–12], strain elastography [13–15] or shear wave elastography [16–18] As reported by Yamaguchi et al. [19, 20] B-mode images of liver parenchyma contain numerous fine echo spots, called “speckle pattern” images [19–21]. Image analysis of speckle patterns can be used to identify tissue characteristics resulting from structural changes.

Acoustic structure quantification (ASQ), proposed by Toyoda et al. [22], uses sonographic software to analyze statistical information about acquired echo signals. The physical principle is that the scatter or deflection of the ultrasound wave when propagating through tissues varies according to the acoustic interfaces it encounters. In normal liver, the scattering fits a Rayleigh distribution [23, 24] because of the presence of many structures smaller than the wavelength of the typical ultrasound wave. By considering the speckle pattern in a region of interest (ROI), quantitative analysis of liver tissue can be used to assess the degree of hepatic fibrosis. Ultrasound wave scattering is increased in fibrotic liver parenchyma compared with normal conditions. ASQ could therefore be a helpful tool in quantifying diffuse liver disease and monitoring regression/progression in patients with liver fibrosis and the effects of fibrosis treatment [25–27].

In this study, we evaluated this quantitative imaging technique through intraoperative measurements of the difference ratio (*C*_m_^2^) of patients with PHT and PP and the degree of gastroesophageal varices and liver function according to Child–Pugh score, aiming to determine the value of ASQ in predicting the severity of PHT.

## Material and methods

### Ethical approval of the study protocol

All subjects included in the study provided written informed consent to participate. The study protocol was approved by the ethics committee of the Air Force Medical University Tangdu Hospital, Xi’an, China.

### Patients and control subjects

The subjects of the study were enrolled from a consecutive series of patients with previously or newly diagnosed PHT who were admitted to the Fourth Military Medical University Tangdu Hospital between April 2010 and January 2012. Patients with hepatocellular carcinoma or intrahepatic thrombosis were excluded from the study. According to the sample size calculation for a two-sample comparison, 60 patients were needed. Patients referred for surgical therapy with portosystemic shunts were enrolled. The indications for shunt surgery in PHT patients include: severe hypersplenism with white blood cell count < 2.0 × 10^9^/L and platelets < 30 × 10^9^/L; and a history of bleeding or severe gastroesophageal varices seen on endoscopy. Subjects in the patient group were further divided into subgroups according to Child–Pugh scoring criteria [28] and the severity of gastroesophageal varices (small/large), which was determined by endoscopic examination as recommended that quantitative size with a suggested cut-off diameter of 5 mm, with large varices being those greater than 5 mm [29].

Thirty healthy volunteers matched with the patients for gender and age were also enrolled. The subjects in the control group had no history of chronic liver disease, pulmonary disease or cardiovascular disease. Laboratory tests showed normal liver function and normal complete blood count.

### ASQ of liver

The patients underwent ultrasound examination on the day before surgery with dedicated equipment and a 5–7 MHz convex probe (Aplio 500; Toshiba Medical Systems, Osaka, Japan) to identify the best acoustic window for ASQ. Each subject was placed in the supine or left lateral position and two images (axial plane and sagittal plane) of the right lobe at intercostal spaces 7 and 8 were obtained. As reported previously, parenchymal vessels and perivascular connective tissue are responsible for marked variation in ASQ findings [22]. The display depth and transmit focus were set to 4 cm. When images showing a high proportion of parenchyma free from large vascular structures and perivascular connective tissue were observed, the “ASQ” button was pressed and the raw data collected and stored for offline processing by the integrated software. Real time images were collected over 3 s, preferably intercostal and subcostal at maximal inspiration.

**Fig. 1:**
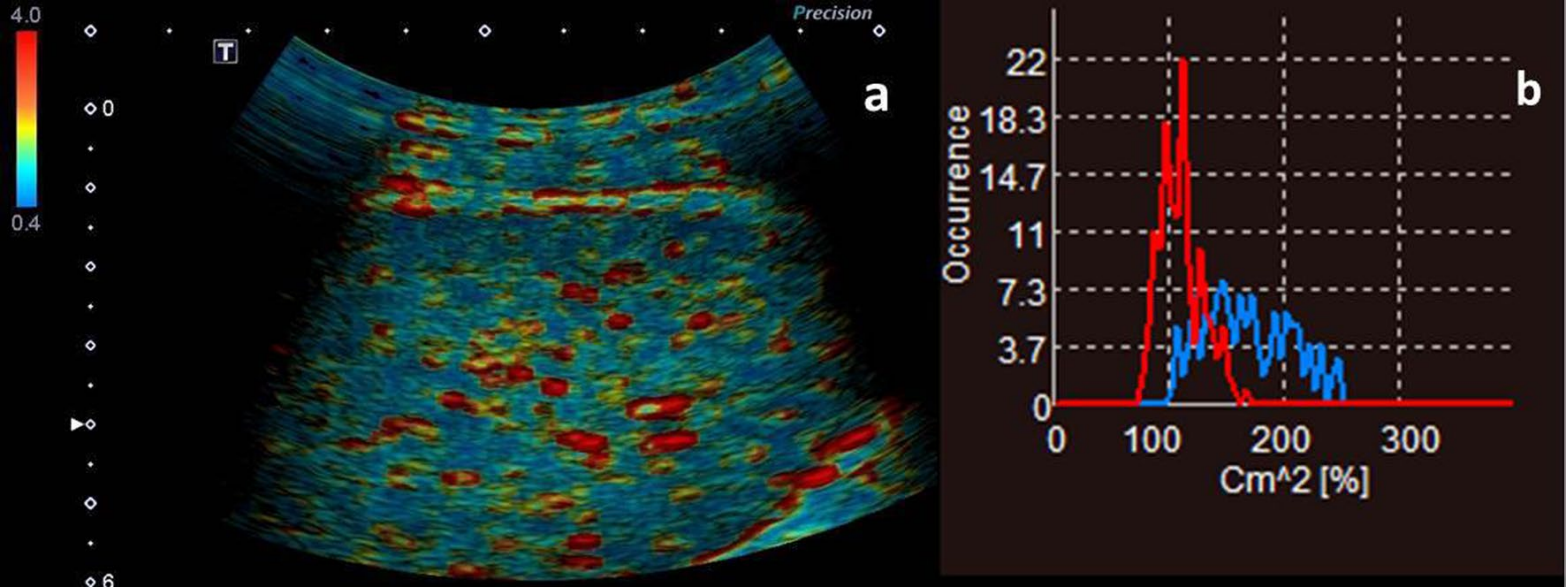
Acoustic structure quantification (ASQ) analysis images obtained from a patient with portal hypertension, right lobe of liver. **a** Histogram display of the right lobe; **b** the corresponding ASQ curve for the difference ratio (*C*_m_^2^). The red curve represents the value of *C*_m_^2^ in the region of interest; higher inhomogeneous inconsistent values are indicated by the blue curve

All images obtained for ASQ analysis were displayed as color-coded maps based on the amplitude of echoes and on the scattering (i.e. the deflection of the ultrasound wave) produced by different tissue interfaces (Fig. 1a). The ROI was positioned at a depth of 4–6 cm depending on the dimensions of the area of the liver that was most free from Glisson’s capsules, as previously reported. The results were displayed on a *C*_m_^2^ histogram (Fig. 1b). The output data of the ASQ analysis were in the form of *C*_m_^2^ values, *C*_m_^2^ being a statistical parameter derived from the distribution of the echo amplitude. It is calculated using the equation $C_{m}^{2} = \frac{{\sigma_{m}^{2} }}{{\sigma_{R}^{2} \left( \mu \right)}} = \left( {\frac{\pi }{4 - \pi }} \right)\frac{{\sigma_{m}^{2} }}{{\mu_{m}^{2} }}$ and can also be expressed as: $C_{{\text{m}}}^{2} = \frac{{{\text{Measured}}\;{\text{Variance}}}}{{{\text{Estimated}}\;{\text{Variance}}\;{\text{of}}\;{\text{normal}}\;{\text{liver}}}}\left[ \% \right]$, where *μ* and *σ*^2^ are the average and variance, respectively, of the echo amplitude in a small ROI and *σ*_m_ is the variance calculated from limited samples of less than *μ* + 4*σ*. *σ*_R_^2^(µ) is the variance of the Rayleigh distribution estimated from the measured average.

The calculated ASQ parameters were: *C*_m_^2^_max_ (the peak value of *C*_m_^2^); *C*_m_^2^_mean_ (the mean value of *C*_m_^2^); and *R*_max_*C*_m_^2^ (the highest occurred *C*_m_^2^ value of the red curve).As an innovative measurement, it is necessary to test the reliability. 10 patients and 10 healthy volunteers were tested by two operators for inter-operator variability and were tested by one operator twice for intra-operator variability. The inter/intra-operator variability were tested by Intraclass Correlation Coefficient analysis.

### Doppler ultrasonography

All subjects underwent conventional abdominal Doppler ultrasonography with a 3.5 MHz transducer (Aplio 500; Toshiba Medical Systems, Osaka, Japan). For ASQ, the probe with a much higher frequency (5–7 MHz) were applied to acquire high resolution imaging in near field, but for conventional B mode scanning, especially for Doppler ultrasound measurement, a low frequency ultrasound with strong penetration was better. Doppler parameters including velocity and resistive index were measured at 30°–60°. The Doppler gate was placed in the porta hepatis and in the hilum of the spleen to measure parameters for the portal vein, hepatic artery and splenic artery. Velocity measurements were made at an angle of 30°–60°. The mean velocity in the portal vein (PVVel), the hepatic artery resistive index (HARI) and the splenic artery resistive index (SpARI) were calculated automatically by the machine after the waveform traces for three cardiac cycles had been obtained. The portal hypertension index (PHI) was calculated as: PHI = (HARI × 0.69)(SpARI × 0.87)/PVVel [30, 31]. They were calculated as the mean of three measurements.

### Measurement of PP

The drugs used for general anesthesia were the same in all subjects. Heart rate, electrocardiogram, oxygen saturation, end-tidal carbon dioxide pressure, blood pressure and temperature were monitored continuously. Intraoperatively, with the blood pressure kept constant, the right gastroepiploic vein was isolated and catheterized with a pressure gauge to measure the PP. An examiner (Ji-Kai Yin) with 8 years of experience of PP measurement during surgery performed all PP studies. The mean value of PP was calculated from three repeated measurements.

### Statistical analysis

Data were presented as the mean ± standard deviation. Differences between the patient group and control group were tested using the unpaired Student’s *t*-tests. As an innovative measurement, the inter/intra-operator variability of ASQ were tested by Intraclass Correlation Coefficient analysis. All ASQ variables were normal distributed. The Tukey–Kramer multiple comparisons was used for assessing whether there was a significant difference of the ASQ parameters among subgroups according to the Child–Pugh scoring and the gastroesophageal varices grade. Spearman’s rank correlation coefficient and linear regression (simple linear model) analysis were used to assess correlations among the ASQ parameters with PP, Child–Pugh score and varices grade, as the last two were semi-quantitative data and ranked data. The predictive performance of the ASQ parameters for PHT diagnose, liver function evaluation and varices grading were tested using receiver operating characteristic (ROC) curve analysis and expressed in terms of accuracy, sensitivity, specificity and Youden’s index (YI) for several cut-off values. Results were considered significant at *P* < 0.05. The statistical software package SPSS 12.0 (SPSS Inc., Chicago, IL) was used for all data analyses.

## Results

### Sample characteristics

Sixty consecutive patients (46 men and 14 women; median age, 47 years) who underwent portosystemic shunt surgery were enrolled in the present study. There was no significant difference between patients and healthy controls (22 males and 8 females; age range, 32–58 years) as far as gender and age were concerned. The main clinical and pathologic data for the patients were presented in Table 1. Hepatitis B was the most common etiologic factor and was present in 40 cases (67%). Hepatitis C was the cause of cirrhosis in 14 patients (23%). Liver function was classified according to Child–Pugh score as follows: A in 17 (28%) patients; B in 37 (62%); and C in six (10%). Most patients (70%) had light ascites. Among the PHT patients enrolled in our study, 55 had a history of bleeding (12 patients had suffered more than one episode; 43 had a single episode). Twelve patients with multiple bleeding history cannot endure endoscopic examination, the extent of gastroesophageal varices was unknown. The rest five patients without a bleeding history were diagnosed with severe hypersplenism and no gastroesophageal varices were seen on endoscopy. Of the patients who had suffered a single episode of bleeding, 35 had large gastroesophageal varices and eight had small gastroesophageal varices on endoscopy. The mean PP measured directly from the right gastroepiploic vein was 28.85 ± 3.134 mm Hg.

**Table 1:** Clinical and pathologic characteristics of 60 patients with PHT

Patient characteristics	
Age (years), median (range)	47 (26–59)
Sex (F/M)	14/46
*Etiology of liver disease*	
Post-hepatitis B	40
Post-hepatitis C	14
Alcoholic	3
Cryptogenic	3
Child–Pugh class (A/B/C)	17/37/6
Ascites (absent/light/moderate/severe)	8/42/8/2
*Gastroesophageal varices*	
Absent	5
Present (small/large)	8/35
Not available	12
PP (mm Hg), mean ± SD	28.85 ± 3.134

### Comparisons of ASQ and Doppler parameters

**Fig. 2:**
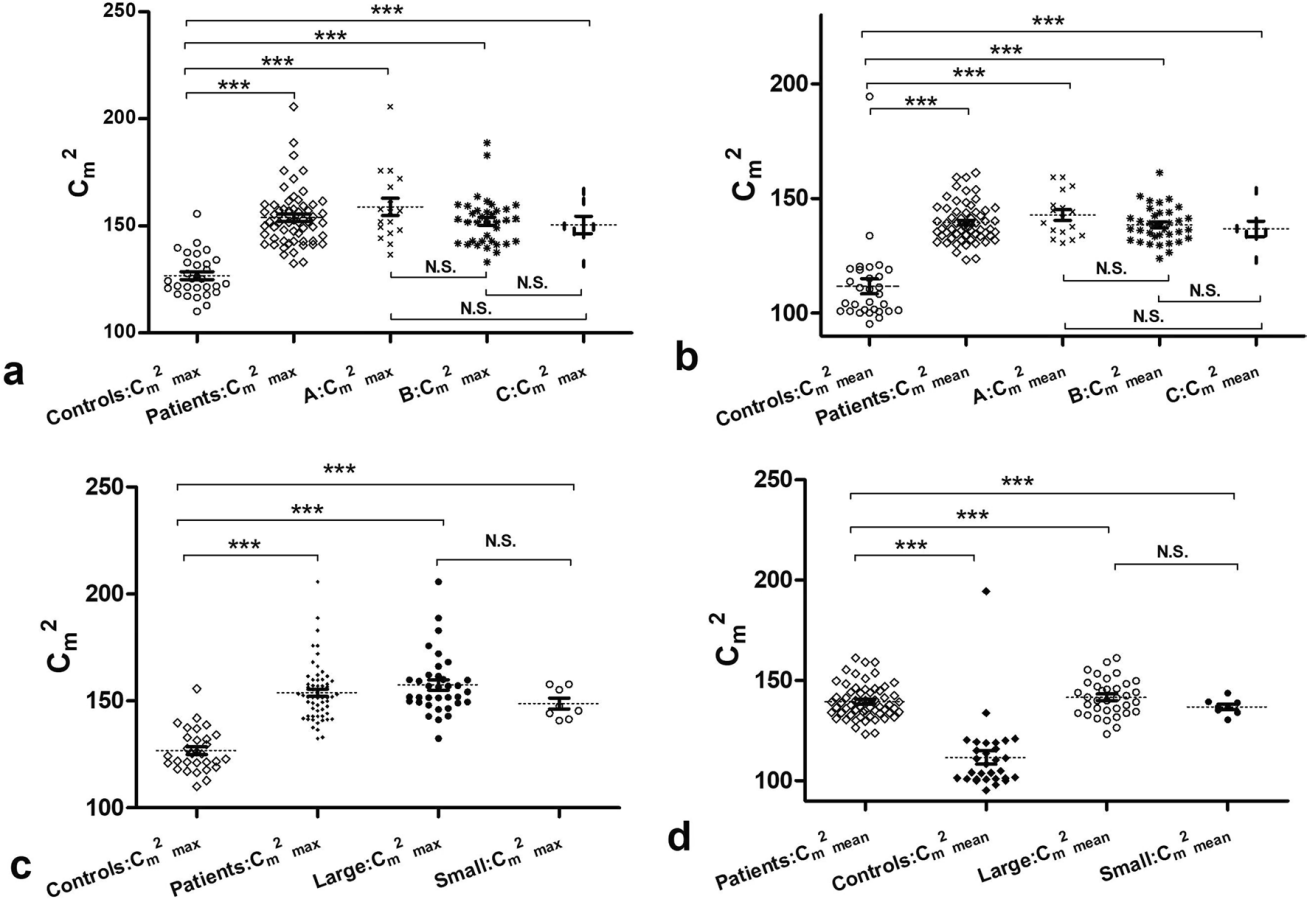
Statistical significance of differences for acoustic structural quantification parameters analyzed by unpaired Student’s *t*-tests or Tukey’s multiple comparison tests as indicated. **a** Peak difference ratio (*C*_m_^2^_max_) with Child–Pugh class; **b** mean difference ratio (*C*_m_^2^_mean_) with Child–Pugh class; **c** *C*_m_^2^_max_ with severity of gastroesophageal varices; **d** *C*_m_^2^_mean_ with severity of gastroesophageal varices. N.S., not significant

As shown in Additional file 1, the ICC of patient R_max_C_m_^2^ were 0.927 (inter-operator variability) and 0.963 (intra-operator variability). That of healthy volunteers R_max_C_m_^2^ were 0.948 (inter-operator variability) and 0.980 (intra-operator variability). The inter/intra-operator variability of ASQ were highly consistent. Comparisons of the Doppler and ASQ parameters among the patients and healthy subjects are shown in Table 2. The ASQ parameters *C*_m_^2^_max_ and *C*_m_^2^_mean_, except *R*_max_*C*_m_^2^ in PHT patients were much higher than those in the healthy subjects, with *P* < 0.01. The Doppler parameter PHI in the patient group was 1.843 ± 0.6205 m s^–1^, which increased greatly compared with the healthy controls. Multiple comparisons indicated that, regardless of Child–Pugh class and degree of gastroesophageal varices, the patients had significantly increased *C*_m_^2^_max_ and *C*_m_^2^_mean_ compared with the controls (all *P* < 0.0001). No differences were observed among the subgroups. The statistical significance of differences in *C*_m_^2^_max_ and *C*_m_^2^_mean_ between groups was shown in Fig. 2.

**Table 2:** Comparison of ASQ and Doppler parameters between healthy subjects and patients

US variables	Healthy Control	Patients with PHT	*P*-value
C_m_^2^_max_	126.8 ± 9.886	153.9 ± 13.30	< 0.0001
C_m_^2^_mean_ R_max_C_m_^2^ PHI(m s^−1^)	111.7 ± 18.12 115.4 ± 8.991 1.434 ± 0.3057	139.5 ± 8.553 119.9 ± 8.511 1.843 ± 0.6205	< 0.0001 0.0272 0.0012

### Correlations of ASQ parameters with PHT severity and Doppler parameters

**Fig. 3:**
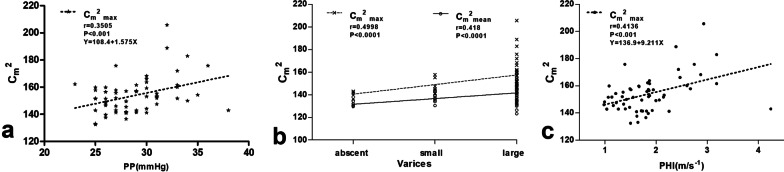
Correlations among acoustic structure quantification parameters, portal hypertension severity and Doppler parameters. Positive relationships were found between **a** peak difference ratio (*C*_m_^2^_max_) and portal pressure (PP); **b** both *C*_m_^2^_max_ and mean difference ratio (*C*_m_^2^_mean_) with degree of varices; and **c** *C*_m_^2^_max_ and the Doppler parameter portal hypertension index (PHI)

**Fig. 4:**
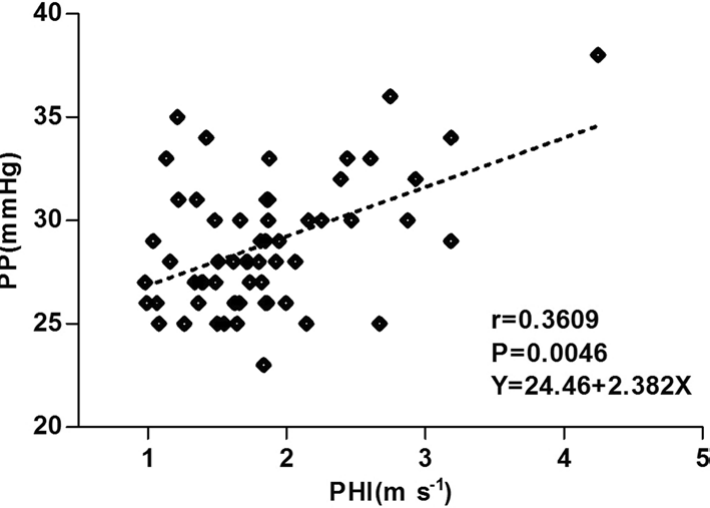
Correlation between the Doppler parameter portal hypertension index (PHI) and portal pressure (PP). PHI was found to be positively correlated with PP

The statistical significance of correlations between ASQ parameters, PHT and Doppler parameters were evaluated using Spearman’s rank correlation coefficient and linear regression analysis, as shown in Fig. 3. In the patients with PHT enrolled in our study, *C*_m_^2^_max_ was significantly statistically correlated with PP (*r* = 0.3505, *P* < 0.01), degree of varices (*r* = 0.4998, *P* < 0.0001) and the Doppler parameter PHI (*r* = 0.4136, *P* < 0.01). *C*_m_^2^_mean_ was significantly correlated with degree of varices only (*r* = 0.4118, *P* < 0.01). *R*_max_*C*_m_^2^ in PHT patients showed no obvious relationship with Child–Pugh scores, varices grade or PHI. None of the ASQ parameters showed a significant correlation with Child–Pugh scores, with *P*-values of 0.1663, 0.0920 and 0.5341 for *C*_m_^2^_max_, *C*_m_^2^_mean_ and *R*_max_*C*_m_^2^, respectively. PHI correlated with PP; namely, at higher PP, increased PHI was observed (*r* = 0.3609, *P* = 0.0046) (Fig. 4), but not with Child–Pugh scores and varices grade.

**Fig. 5:**
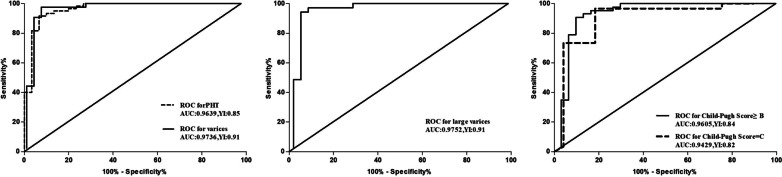
Receiver operating characteristic curves for peak difference ratio (*C*_m_^2^_max_) in diagnosing portal hypertension (PHT) and evaluating PHT severity

Given the above statistical results, the predictive performance of *C*_m_^2^_max_, as the most ideal parameter, was analyzed. The diagnostic YI values for *C*_m_^2^_max_ with a cut-off value of 140.3 in predicting the presence of PHT, gastroesophageal varices and liver function equal to or worse than Child–Pugh B were 0.85 (sensitivity, 91.67%; specificity, 93.33%), 0.91 (sensitivity, 97.67%; specificity, 93.33%) and 0.84 (sensitivity, 90.70%; specificity, 93.33%), respectively. For predicting the presence of a large area of gastroesophageal varices, the diagnostic cut-off value was 142.4 and the corresponding YI was 0.91 (sensitivity, 94.29%; specificity, 96.67%). In PHT patients with Child–Pugh C liver function, where *C*_m_^2^_max_ was 132.3, YI reached its highest value of 0.73. The predictive performance including the area under the curve, sensitivity, specificity and YI with corresponding cut-off values is summarized in Table 3. ROC curves are shown in Fig. 5.

**Table 3:** Predictive performance of *C*_m_^2^_max_ for the assessment of PHT severity

	AUC^a^	Std. Error	*P*-value	95% CI^b^	Cut-off	Sensitivity (%)	Specificity (%)	YI
Presence of PHT	0.9639	0.0217	< 0.0001	0.9213 to 1.006	140.3	91.67	93.33	0.85
Varices	0.9736	0.0197	< 0.0001	0.9350 to 1.012	140.3	97.67	93.33	0.91
Large area of varices	0.9752	0.0188	< 0.0001	0.9383 to 1.012	142.4	94.29	96.67	0.91
^c^Child-Pugh grade ≥ B	0.9605	0.0240	< 0.0001	0.9134 to 1.008	140.3	90.70	93.33	0.84
Child–Pugh grade = C	0.9429	0.0427	0.0003	0.8591 to 1.027	144.0	96.67	85.71	0.82

^a^AUC, area under ROC curve

^b^CI, confidence interval

^c^Child-Pugh class ≥ B means that liver function according to the Child–Pugh score was equal to or worse than the Child–Pugh B

## Discussion

Increased PP in patients with chronic liver disease leads to a high risk of developing gastroesophageal varices and decompensation of liver function. Because gastroesophageal varices-induced hemorrhage in patients with cirrhosis can be fatal, these patients must be routinely classified according to their risk status and appropriate therapy should be undertaken to prevent hemorrhage. Endoscopy is the gold standard method for screening for esophageal varices in cirrhotic patients; however, the extent of esophageal varices can vary and follow-up for variceal bleeds is needed, which limits its routine application. HVPG is an acceptable indirect measurement of PHT and predictor of the complications of PHT. It is a strong and independent predictor of death in both compensated and decompensated cirrhosis [32, 33]. Variceal bleeding does not occur at pressures under 12 mm Hg as indicated by HVPG. No noninvasive method is available to replace HVPG measurement or endoscopy in the rapid discrimination of stage of liver cirrhosis and post-treatment evaluation, and we remain in need of noninvasive means to predict the presence or absence of gastroesophageal varices.

Histology is an important indicator of the severity of chronic liver disease and of the degree of PHT. One method for the histologic evaluation of the liver is measurement of liver stiffness by ultrasound elastography. Much research has been conducted in this field and some techniques have been applied in routine clinical work to assist the evaluation of PHT. The guidelines published in 2018 pointed out that liver stiffness measurements of TE > 20 kPa can be used to identify patients likely bearing clinically significant portal hypertension (HVPG ≥ 10 mmHg); liver stiffness measurement using TE < 20–25 kPa combined with platelet count > 110–150 × 106/mL is useful in ruling out varices needing treatment [34]. In the present study, ASQ technology based on B-mode ultrasonographic images, obtained through conventional B-mode ultrasonography, was used to identify homogeneous and heterogeneous liver changes not subjectively but objectively. It can be difficult for sonographers to make quantitative diagnoses on conventional ultrasonography as is possible with computed tomography. ASQ software analyzes the physical phenomenon of ultrasound wave scattering and by measuring the degree of scattering provides a quantitative assessment of liver parenchymal histology.

We acquired real time images over 3 s depicting liver parenchyma free from Glisson’s capsules with the patient holding his or her breath. Three display modes are available in ASQ analysis: histogram, parametric and basic. Histogram mode gives the *C*_m_^2^ value after analyzing the raw data; we acquired 22 consecutive frames for each patient. We chose three parameters as indicators of liver histology: *C*_m_^2^_max_, *C*_m_^2^_mean_ and *R*_max_*C*_m_^2^, representing the peak, average and highest occurred value of *C*_m_^2^ of the obtained red curve in the ROI, respectively. The final values we used for statistical analysis were the means of those from each of the 22 frames. Parametric mode provides images colored according to echo amplitude, with *C*_m_^2^ values coded from red to green. Basic mode provides another parameter, the probability density function of the *C*_m_^2^ values measured in a ROI, displayed against the theoretical speckle pattern generated by a Rayleigh distribution [27]. In the present study, we focused on histogram analysis.

Three clinical indices – PP measured intraoperatively, the size of gastroesophageal varices and liver function classified by Child–Pugh score—were used as indicators of the severity of PHT [35–37]. The ASQ parameters were significantly increased in patients with PHT; *C*_m_^2^_max_ was better correlated with PP and the size of gastroesophageal varices than *C*_m_^2^_mean_ and *R*_max_*C*_m_^2^. C_m_^2^_max_ was also positively correlated with the index of hemodynamic change PHI, which increased significantly with increased PP. *C*_m_^2^_mean_ was correlated only with the size of gastroesophageal varices. No obvious relationships were found between the ASQ parameters and Child–Pugh class. According to ROC analysis, the diagnostic value of *C*_m_^2^_max_ for PP, severity of varices and liver function was greater than that of the ASQ parameters for liver fibrosis [26]. We also evaluated hemodynamic changes in patients with PHT and their relationship with liver pathologic changes. PHI, which was significantly correlated with PP in our previous studies [38], showed a good correlation with PP and with *C*_m_^2^_max_. To our knowledge, this is the first study to evaluate this relationship with regard to histology, hemodynamics and pressure simultaneously.

Our study had limitations. First, we did not assess the middle stage between normal liver and decompensated liver cirrhosis (e.g. liver fibrosis and liver cirrhosis without PHT) due to the impossibility to get the data of portal pressure of the middle stage and this may be the reason for the high diagnostic value of *C*_m_^2^_max_. Second, the number of patients was not large enough for a comprehensive evaluation, especially of varices, which might be the reason of a week relationship (r = 0.3 ~ 0.4) to be found between these parameters. Further studies are needed to confirm the present results regarding the predictive value of ASQ parameters.

We evaluated correlations between ultrasonographic images and the histologic changes of liver parenchyma, hepatic dynamic changes, PP, gastroesophageal varices and Child–Pugh class. We employed ASQ analysis in histogram mode because the parameters were most stable in this mode. Our results show that the predictive performance of *C*_m_^2^_max_ was satisfactory and warrants further validation. We can infer that ASQ analysis of ultrasound images may have a role in the evaluation of the severity of PHT by identifying liver histologic changes from the speckle pattern observed in cirrhosis.

## Supplementary Information

**Additional file 1. Suppl. Fig 1.** Intra-operator variability of patients: RmaxCm2 of patients were tested by the same operator on the same patients for two times. **Suppl. Fig 2.** Inter-operator variability of patients: RmaxCm2 of patients were tested by two operators on the same patients. **Suppl. Fig 3.** Intra-operator variability of healthy volunteers: RmaxCm2 of healthy volunteers were tested by the same operator on the same healthy volunteers for two times. **Suppl. Fig 4.** Inter-operator variability of healthy volunteers: RmaxCm2 of healthy volunteers were tested by two operators on the same healthy volunteers.

## Data Availability

All data generated or analysed during this study are included in this published article.

## Abbreviations

ASQ: Acoustic structure quantification
PHT: Portal hypertension
PP: Portal pressure
HVGP: Hepatic venous pressure gradient
TE: Transient elastography
SE: Strain elastography
SWE: Shear wave elastography
C_m_^2^_max_: Peak value of C_m_^2^
C_m_^2^_mean_: Mean value of C_m_^2^
R_max_C_m_^2^: C_m_^2^ value at the peak red occurrence
PVVel: Mean velocity of the portal vein
HARI: Hepatic artery resistive index
SpARI: Splenic artery resistive index
PHI: Portal hypertension index
ROC: Receiver operating characteristic
PDF: Probability density function
ROI: Region of interest
CI: Confidence interval
YI: Youden’s index

## References

[1] Gunarathne LS, Rajapaksha H, Shackel N, Angus PW, Herath CB (2020). Cirrhotic portal hypertension: from pathophysiology to novel therapeutics. World J Gastroenterol.

[2] Suk KT (2014). Hepatic venous pressure gradient: clinical use in chronic liver disease. Clin Mol Hepatol.

[3] Snowdon VK, Fallowfield JA (2016). Editorial: measuring inflammatory and fibrotic components of portal hypertension—a noninvasive hepatic venous pressure gradient?. Aliment Pharmacol Ther.

[4] Bochnakova T (2021). Hepatic venous pressure gradient. Clin Liver Dis (Hoboken).

[5] Shao R, Li Z, Wang J, Qi R, Liu Q, Zhang W, Mao X, Song X, Li L, Liu Y (2020). Hepatic venous pressure gradient-guided laparoscopic splenectomy and pericardial devascularisation versus endoscopic therapy for secondary prophylaxis for variceal rebleeding in portal hypertension (CHESS1803): study protocol of a multicenter randomised controlled trial in China. BMJ Open.

[6] Iwakiri Y, Groszmann RJ (2006). The hyperdynamic circulation of chronic liver diseases: from the patient to the molecule. Hepatology.

[7] Bolognesi M, Di Pascoli M, Sacerdoti D (2017). Clinical role of non-invasive assessment of portal hypertension. World J Gastroenterol.

[8] Baik SK, Kim JW, Kim HS, Kwon SO, Kim YJ, Park JW, Kim SH, Chang SJ, Lee DK, Han KH (2006). Recent variceal bleeding: Doppler US hepatic vein waveform in assessment of severity of portal hypertension and vasoactive drug response. Radiology.

[9] Baik SK (2010). Haemodynamic evaluation by Doppler ultrasonography in patients with portal hypertension: a review. Liver Int.

[10] Effenberger M, Grander C, Fritsche G, Bellmann-Weiler R, Hartig F, Wildner S, Seiwald S, Adolph TE, Zoller H, Weiss G (2020). Liver stiffness by transient elastography accompanies illness severity in COVID-19. BMJ Open Gastroenterol.

[11] Rockey DC (2008). Noninvasive assessment of liver fibrosis and portal hypertension with transient elastography. Gastroenterology.

[12] Friedrich-Rust M, Ong MF, Martens S, Sarrazin C, Bojunga J, Zeuzem S, Herrmann E (2008). Performance of transient elastography for the staging of liver fibrosis: a meta-analysis. Gastroenterology.

[13] Fang C, Virdee S, Jacob J, Rufai O, Agarwal K, Quaglia A, Quinlan DJ, Sidhu PS (2019). Strain elastography for noninvasive assessment of liver fibrosis: a prospective study with histological comparison. Ultrasound.

[14] Friedrich-Rust M, Ong MF, Herrmann E, Dries V, Samaras P, Zeuzem S, Sarrazin C (2007). Real-time elastography for noninvasive assessment of liver fibrosis in chronic viral hepatitis. AJR Am J Roentgenol.

[15] Havre RF, Elde E, Gilja OH, Odegaard S, Eide GE, Matre K, Nesje LB (2008). Freehand real-time elastography: impact of scanning parameters on image quality and in vitro intra- and interobserver validations. Ultrasound Med Biol.

[16] Ferraioli G, Barr RG (2020). Ultrasound liver elastography beyond liver fibrosis assessment. World J Gastroenterol.

[17] Sporea I, Sirli R, Bota S, Popescu A, Sendroiu M, Jurchis A (2012). Comparative study concerning the value of acoustic radiation force impulse elastography (ARFI) in comparison with transient elastography (TE) for the assessment of liver fibrosis in patients with chronic hepatitis B and C. Ultrasound Med Biol.

[18] Sporea I, Bota S, Peck-Radosavljevic M, Sirli R, Tanaka H, Iijima H, Badea R, Lupsor M, Fierbinteanu-Braticevici C, Petrisor A (2012). Acoustic Radiation Force Impulse elastography for fibrosis evaluation in patients with chronic hepatitis C: an international multicenter study. Eur J Radiol.

[19] Yamaguchi T, Hachiya H, Kamiyama N, Moriyasu F (2002). Examination of the spatial correlation of statistics information in the ultrasonic echo from diseased liver. Jpn J Appl Phys.

[20] Yamaguchi T, Hachiya H, Kamiyama N, Ikeda K, Moriyasu N (2001). Estimation of characteristics of echo envelope using RF echo signal from the liver. Jpn J Appl Phys.

[21] Kamiyama N, Yamaguchi T, Hachiya H (2003). Tissue characterization using statistical information from ultrasound echo signals. Med Imag Technol.

[22] Toyoda H, Kumada T, Kamiyama N, Shiraki K, Takase K, Yamaguchi T, Hachiya H (2009). B-mode ultrasound with algorithm based on statistical analysis of signals: evaluation of liver fibrosis in patients with chronic hepatitis C. AJR Am J Roentgenol.

[23] Tsui PH, Zhou Z, Lin YH, Hung CM, Chung SJ, Wan YL (2017). Effect of ultrasound frequency on the Nakagami statistics of human liver tissues. PLoS ONE.

[24] Tuthill TA, Sperry RH, Parker KJ (1988). Deviations from Rayleigh statistics in ultrasonic speckle. Ultrason Imaging.

[25] Cheng L, Chen Y, Xiao R, Pan Y, Guo J (2019). Evaluation of hepatic fibrosis by ultrasonic acoustic structure quantification. Medicine (Baltimore).

[26] Ricci P, Marigliano C, Cantisani V, Porfiri A, Marcantonio A, Lodise P, D'Ambrosio U, Labbadia G, Maggini E, Mancuso E (2013). Ultrasound evaluation of liver fibrosis: preliminary experience with acoustic structure quantification (ASQ) software. Radiol Med.

[27] Kuroda H, Kakisaka K, Kamiyama N, Oikawa T, Onodera M, Sawara K, Oikawa K, Endo R, Takikawa Y, Suzuki K (2012). Non-invasive determination of hepatic steatosis by acoustic structure quantification from ultrasound echo amplitude. World J Gastroenterol.

[28] Tsoris A, Marlar CA: Use of the child pugh score in liver disease. In: *StatPearls.* edn. Treasure Island (FL); 2022. 31194448

[29] Garcia-Tsao G, Sanyal AJ, Grace ND, Carey WD (2007). Practice Guidelines Committee of American Association for Study of Liver D, Practice Parameters Committee of American College of G: prevention and management of gastroesophageal varices and variceal hemorrhage in cirrhosis. Am J Gastroenterol.

[30] Iwao T, Toyonaga A, Oho K, Tayama C, Masumoto H, Sakai T, Sato M, Tanikawa K (1997). Value of Doppler ultrasound parameters of portal vein and hepatic artery in the diagnosis of cirrhosis and portal hypertension. Am J Gastroenterol.

[31] Piscaglia F, Donati G, Serra C, Muratori R, Solmi L, Gaiani S, Gramantieri L, Bolondi L (2001). Value of splanchnic Doppler ultrasound in the diagnosis of portal hypertension. Ultrasound Med Biol.

[32] Merkel C, Bolognesi M, Sacerdoti D, Bombonato G, Bellini B, Bighin R, Gatta A (2000). The hemodynamic response to medical treatment of portal hypertension as a predictor of clinical effectiveness in the primary prophylaxis of variceal bleeding in cirrhosis. Hepatology.

[33] Zipprich A, Garcia-Tsao G, Rogowski S, Fleig WE, Seufferlein T, Dollinger MM (2012). Prognostic indicators of survival in patients with compensated and decompensated cirrhosis. Liver Int.

[34] Ferraioli G, Wong VW, Castera L, Berzigotti A, Sporea I, Dietrich CF, Choi BI, Wilson SR, Kudo M, Barr RG (2018). Liver ultrasound elastography: an update to the world federation for ultrasound in medicine and biology guidelines and recommendations. Ultrasound Med Biol.

[35] Brandenburger LA, Regenstein FG (2002). Variceal hemorrhage. Curr Treat Options Gastroenterol.

[36] Seewald S, Seitz U, Yang AM, Soehendra N (2001). Variceal bleeding and portal hypertension: still a therapeutic challenge?. Endoscopy.

[37] Maruyama H, Yokosuka O (2017). Ultrasonography for noninvasive assessment of portal hypertension. Gut Liver.

[38] Zhang L, Duan YY, Li JM, Yin JK (2007). Hemodynamic features of Doppler ultrasonography in patients with portal hypertension: intraoperative direct measurement of portal pressure in the portal venous system. J Ultrasound Med.

