# Supplementary material for: The severity of portal hypertension by a non-invasive assessment: acoustic structure quantification analysis of liver parenchyma
Source: BMC Med Imaging. 2022 May 12;22:85. doi: 10.1186/s12880-022-00817-2 (PMC9097305; doi:10.1186/s12880-022-00817-2)
Supplement: Supplementary file 1 — Additional file 1. Suppl. Fig 1. Intra-operator variability of patients: RmaxCm2 of patients were tested by the same operator on the same patients for two times. Suppl. Fig 2. Inter-operator variability of patients: RmaxCm2 of patients were tested by two operators on the same patients. Suppl. Fig 3. Intra-operator variability of healthy volunteers: RmaxCm2 of healthy volunteers were tested by the same operator on the same healthy volunteers for two times. Suppl. Fig 4. Inter-operator variability of healthy volunteers: RmaxCm2 of healthy volunteers were tested by two operators on the same healthy volunteers. [file 12880_2022_817_MOESM1_ESM.docx]

The inter/intra-operator variability test.


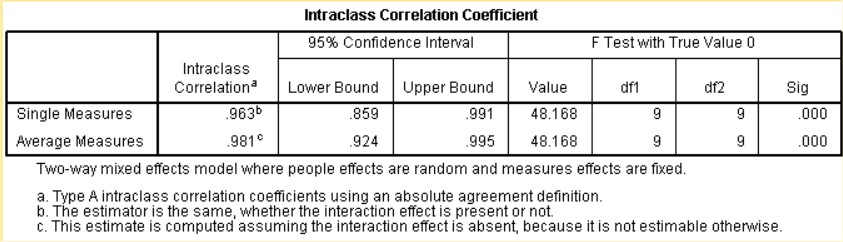


Intra-operator variability of patients: R_max_C_m_^2^ of patients were tested by the same operator on the same patients for two times.


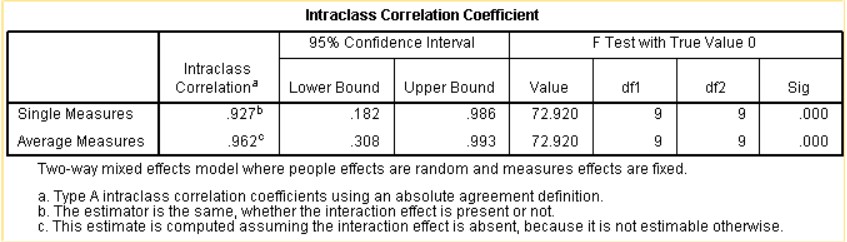


Inter-operator variability of patients: R_max_C_m_^2^ of patients were tested by two operators on the same patients.


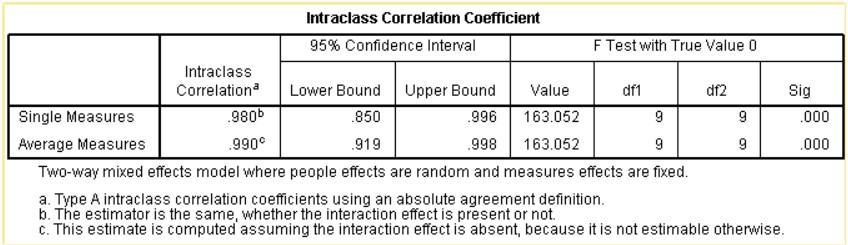


Intra-operator variability of healthy volunteers: R_max_C_m_^2^ of healthy volunteers were tested by the same operator on the same healthy volunteers for two times.


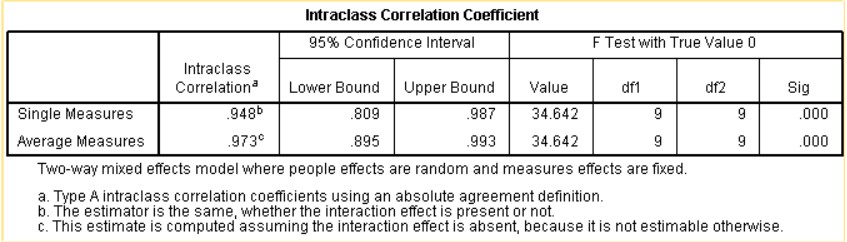


Inter-operator variability of healthy volunteers: R_max_C_m_^2^ of healthy volunteers were tested by two operators on the same healthy volunteers.
